# Diurnal heat stress reduces pig intestinal integrity and increases endotoxin translocation

**DOI:** 10.1093/tas/txx003

**Published:** 2018-01-25

**Authors:** Nicholas K Gabler, Dawn Koltes, Simone Schaumberger, G Raj Murugesan, Nicole Reisinger

**Affiliations:** 1 Department of Animal Science, Iowa State University, Ames, IA; 2 BIOMIN Holding GmbH, Getzersdorf, Austria; 3 BIOMIN America Inc., San Antonio, TX; 4 BIOMIN Research Center, Tulln, Austria

**Keywords:** endotoxin, heat stress, intestinal integrity, pigs

## Abstract

Heat stress negatively affects performance and intestinal integrity of pigs. The objective of this study was to characterize the effects of diurnal heat stress (**dHS**) on nursery-grower pig performance, intestinal integrity, and lipopolysaccharide (**LPS**) translocation. Forty-eight nursery-grower gilts, individually penned, were randomly assigned to two treatments. Twenty-four pigs were then exposed to dHS for 3 d, 6 h at 38°C and 18 h at 32°C, at 40–60% humidity. The remaining pigs were maintained under thermal neutral (**TN**) conditions. Changes in pig rectal temperatures (**Tr**), respiration rates (**RR**), performance, and blood parameters were evaluated. Additionally, ex vivo ileum integrity was assessed with the Ussing chamber by measuring transepithelial resistance (**TER**), and 4 kDa fluorescein isothiocyanate (**FITC**)–dextran (**FD4**) and FITC–LPS mucosal to serosal flux. As expected, dHS increased pig Tr and RR (*P* < 0.05) and reduced pig performance (*P* < 0.05) on the 3-d period. Compared with TN, ileum TER (*P* = 0.04), FITC–LPS (*P* < 0.001), and FD4 (*P* = 0.011) permeability were significantly increased due to dHS. Compared with TN pigs, dHS increased serum endotoxin by 150% (*P* = 0.031). Altogether, 3-d dHS significantly reduced pig performance and intestinal integrity and increased blood endotoxin concentrations.

## INTRODUCTION

Heat stress is a physiological condition resulting from an animal’s inability to regulate their internal euthermic temperature (Baumgard et al., 2012). When pigs are subjected to elevated environmental temperatures and heat indexes (when relative humidity is factored in with the actual air temperature), as seen in summer months and tropical regions, it can be detrimental to performance, health, and wellbeing, and if severe enough even leads to death (St-Pierre et al., 2003; Renaudeau et al., 2011; Baumgard et al., 2012). Without heat abatement strategies, growing pigs are particularly susceptible to heat stress (Renaudeau et al., 2010; Johnson et al., 2015a). As a result, heat stress can modify pre- and post-absorptive metabolism and tissue accretion in growing pigs (Cruzen et al., 2015a; Johnson et al., 2015a; Morales et al., 2016a). These changes are probably linked to reductions in feed intake and intestinal integrity, increases in circulating concentrations of endotoxin, and increases in cellular stress (heat shock and hypoxia response) and oxidative stress markers (Pearce et al., 2014; Liu et al., 2017).

In recent years, many studies in pigs have used constant elevated heat to assess how heat stress alters animal physiology (Pearce et al., 2013a; Boddicker et al., 2014; Sanz Fernandez et al., 2014; Johnson et al., 2015b; Morales et al., 2016a). The major organ first affected by heat stress is the gastrointestinal tract due to the redistribution of blood to the extremities to support heat loss (Lambert et al., 2002). As a result, intestinal function and integrity are reduced, and this can increase the risk of acute endotoxemia within 2–6 h of heat stress exposure in pigs (Pearce et al., 2012, 2013b, 2013c). Endotoxin is synonymously known as lipopolysaccharide (**LPS**) and, in pigs, LPS is a potent immune stimulator that induces inflammation and antagonizes protein synthesis (Webel et al., 1997; Kimball et al., 2003).

Interestingly, there are a few studies in pigs (Patience et al., 2005; Johnson and Lay, 2017; Liu et al., 2017) that have adopted a diurnal heat stress (**dHS**) model to mimic more closely sub-tropical, temperate summer heat cycles. In both cases, heat stress negatively affects performance and intestinal integrity of pigs. Therefore, the objective of this study was to characterize the effects of 3-d dHS on nursery-grower pig performance, intestinal integrity, and endotoxemia.

## MATERIALS AND METHODS

All procedures were reviewed and approved by the Iowa State University Institutional Animal Care and Use Committee (IACUC # 1-14-7704-S).

### Animals and study design

Forty-eight 1-wk post-weaned crossbred gilts (5.2 ± 0.59 kg body weight [**BW**]), consisting of Genetiporc 6.0 × Genetiporc F25 genetics (PIC, Inc., Hendersonville, TN), were assigned to individual pens across two rooms at the Iowa State University Swine Nutrition Farm (Ames, IA, USA). These rooms were maintained at thermal neutral (**TN**) conditions (28°C; 40–60% humidity). All pigs were fed an iso-energetic and iso-nitrogenous diet formulated to meet or exceed the predicted requirements (NRC, 2012) for energy, essential amino acids, proteins, minerals, and vitamins (Table 1).

**Table 1.** Diet composition, as-fed basis

Ingredient	%
Corn, yellow dent	56.54
Corn DDGS	5.00
Soybean meal, 46.5% CP	31.57
Soybean oil	1.00
Salt	0.50
Optiphos 2000	0.02
Vitamin-mineral pre-mix^†^	0.30
Limestone	1.23
Monocalcium phosphate 21%	0.73
dl-Methionine	0.11
l-Threonine	0.09
l-Lysine HCl	0.40
Fish meal, menhaden	2.50
Calculated composition	
ME, kcal/kg.	3360
Crude protein, %	23.0
SID lysine, %	1.25

SID, standardized ileal digestibility.

^†^Premix supplied (per kg of diet): 8,820 IU vitamin A, 1,653 IU vitamin D3, 33.1 IU vitamin E, 4.4 mg vitamin K, 6.6 mg riboflavin, 38.9 mg niacin, 22.1 mg pantothenic acid, 0.04 mg vitamin B12, 1.1 mg I as potassium iodide, 0.30 mg Se as sodium selenite, 60.6 mg Zn as zinc oxide, 36.4 mg Fe as ferrous sulfate, 12.1 mg Mn as manganous oxide, and 3.6 mg Cu as copper sulfate.

In six replicates and after a 21–28 d acclimation period, pigs (~21 kg BW) were then subjected to a 3-d environmental challenge period. One room (*n* = 24 individually penned pigs) were exposed to dHS (for 6 h at 38°C at 40–60% humidity and for 18 h under upper thermal neutral conditions of 32°C at 40–60% humidity). The remaining room of 24 pigs was maintained in individual pens for these 3 d under TN conditions (28°C at 40–60% humidity). Each room’s temperature and humidity were continuously monitored and recorded every 5 min by a data recorder (Lascar model EL-USB-2-LCD, Erie, PA). All pen spacing, feeders, and waterers were identical in both rooms.

At day 0, 28, and 31, all pigs were weighed and feed disappearance recorded. Days 0–28 represented the pre-challenge period, whereas days 29–31 represented the environmental challenge period. During the environmental challenge period, body temperature indices were obtained via rectal temperature (**Tr**) every 2 h for the 6-h period using a standard digital thermometer (ReliOn, Waukegan, IL) and respiration rates (**RR**) were determined by counting flank movements equated to breaths per minute.

### Sample collection

At the end of the 3-d environmental challenge period, blood samples were taken from all pigs, before they were sacrificed. Via jugular venipuncture, blood was collected (10 ml) into vacutainer tubes (BD, Franklin Lakes, NJ), clotted, and centrifuged at 2,500 × *g* for 10 min at 4°C. Serum was then obtained, aliquoted, and stored at −80°C for later analysis. Then, pigs were sacrificed via captive bolt gun and exsanguination. Proximal ileum tissue sections (~1.5 m before the ileal–cecal junction) were immediately harvested following euthanasia. Fresh segments of whole ileum were flushed of luminal contents, placed immediately into Krebs–Henseleit buffer (**KHB**; containing 25 mM NaHCO_3_, 120 mM NaCl, 1 mM MgSO_4_, 6.3 mM KCl, 2 mM CaCl, and 0.32 mM NaH_2_PO_4_, pH 7.4) under constant aeration, and transported to the laboratory for mounting into Ussing chambers. In addition, ileum and *Longissimus dorsi* (**LD**) muscle tissue samples were snap-frozen in liquid nitrogen and stored at −80°C until later analysis.

### Blood analysis

Serum was analyzed for endotoxin, LPS-binding protein (**LBP**), glucose, non-esterified fatty acids (**NEFA**), blood urea nitrogen (**BUN**), insulin, tumor-necrosis factor-α (**TNF-α**), interleukin 1-β (**IL-1β**), and haptoglobin using commercially available kits and as described previously (Pearce et al., 2015; Schweer et al., 2016; Curry et al., 2017). Briefly, insulin was analyzed in duplicate using an ELISA kit solid-phase two-site enzyme immunoassay based on the sandwich technique (Mercodia Porcine Insulin ELISA, ALPCO Diagnostics, Salem, NH). The assay was conducted in 96-well microplates and read at 450 nm using a Synergy 4 microplate reader (Bio-Tek, Winooski, VT). NEFA (Wako HR Series NEFA-HR, Wako Diagnostics, Richmond, VA), BUN (Quantichrom Urea Assay Kit, BioAssay Systems, Hayward, CA), TNF-α, and IL-1β (R&D systems, Minneapolis, MN) concentrations were measured using commercially available assay kits. Serum haptoglobin (ALPCO Diagnostics, Salem, NH) and LBP (Hycult Biotech, Plymouth Meeting, PA) concentrations were determined by ELISA kits. Serum endotoxin concentrations were determined in triplicate using a recombinant Factor C (rFC) endotoxin assay with a 1/1,000 dilution factor for porcine plasma samples (PyroGene Recombinant Factor C Endotoxin Detection System, Lonza, Walkersville, MD). The plates were then read under fluorescence using a Synergy 4 microplate reader (Bio-Tek, Winooski, VT) with excitation/emission wavelength of 380/440 nm. Endotoxin concentrations (RFU) were expressed as arbitrary units. Serum secretory phospholipase A2 (**sPLA2**) activity was determined using a Cayman Chemical (Ann Arbor, MI) assay kit and data are reported as µmol/min/ml.

### Ex vivo intestinal integrity measures

Freshly isolated ileum segments from each animal were mounted into modified Easy Mount Ussing chambers (Physiological Instruments, San Diego, CA and World Precision Instruments, New Haven, CT) for determination of intestinal integrity and macromolecule transport as described previously (Mani et al., 2013; Pearce et al., 2013b,  2013c). Tissue samples were pinned and placed vertically into the chambers, with the mucosal membrane facing one-half of the chamber and the serosal membrane facing the other half. Each side of the membrane was bathed in 4 ml of KHB, and tissue was treated with a constant O_2_–CO_2_ mixture. Individual segments were then voltage clamped (0 mV) and, after 20-min stabilization, transepithelial electrical resistance (**TER**) was calculated by averaging the current during the first 20 min of tissue stabilization (Gabler et al., 2007). Then, macromolecule and LPS mucosal to serosal permeability was assessed in different ileum sections using 4.4-kDa fluorescein isothiocyanate-labeled-dextran (**FD4**) and fluorescein isothiocyanate-labeled-LPS (**FITC-LPS**) obtained from Sigma-Aldrich (St. Louis, MO). Samples from the serosal side were obtained every 20 min for 120 min, read with a fluorescence spectrophotometer (495 nm excitation), and an apparent permeability coefficient was calculated; FD4 or FITC–LPS flux = d*Q*/(d*t* × *A* × *C*_0_), where d*Q*/d*t* = transport rate (µg/min); *C*_0_ = initial concentration in the donor chamber (µg/ml); *A* = area of the membrane (cm^2^).

### Protein abundance

Western blot analysis was performed on frozen ileum and LD tissue for protein abundance of heat shock protein 70 (HSP 70) and hypoxia-inducible factor 1-α (HIF 1-α). Briefly, whole tissue protein from 500 mg of ileum and LD tissue was extracted, and semi-quantitative protein abundance of HSP70 and HIF-1α were determined as described previously (Pearce et al., 2013b,  2014). Equivalent protein (15 µg) from each sample was then loaded into the lanes of the gel and the proteins were separated by 10% (SDS–PAGE). Membranes were blocked for 1 h in 5% non-fat dry milk (**NFDM**) in TBST (1× TBS, 0.1% Tween-20) and then blocked in primary antibody with 5% NFDM in TBST overnight. After blocking in primary antibody (HSP 70 and HIF 1-α), membranes were incubated in secondary antibody for 1 h. For detection, Supersignal West Pico Chemiluminescent Substrate was used (Thermoscientific, Waltham, MA). Membranes were then imaged using FOTO Analyst Luminary/FX (Fotodyne Inc., Hartland, WI) and bands quantified by densitometry using TotalLab Quant (Total Lab, Newcastle Upon Tyne, UK). A pooled reference sample was run on each gel and used to normalize across gels.

### Statistical analysis

Pig was the experimental unit and all data were statistically analyzed using the PROC MIXED procedure of SAS version 9.2 (SAS Inst. Inc., Cary NC). The model included the fixed effect of TN and dHS. No significant effects were observed between replicates and therefore not included in the model. The 3-d repeated measurements of Tr and RR from each animal were analyzed using repeated measures with time as the repeated effect. The repeated-measures model utilized fixed effects of treatment (TN and dHS), time, and the treatment by time interaction. All data are reported as least square means and considered significant if *P* ≤ 0.05 and a tendency if *P* ≤ 0.10.

## RESULTS

### Phenotypic response

**Figure 1.**
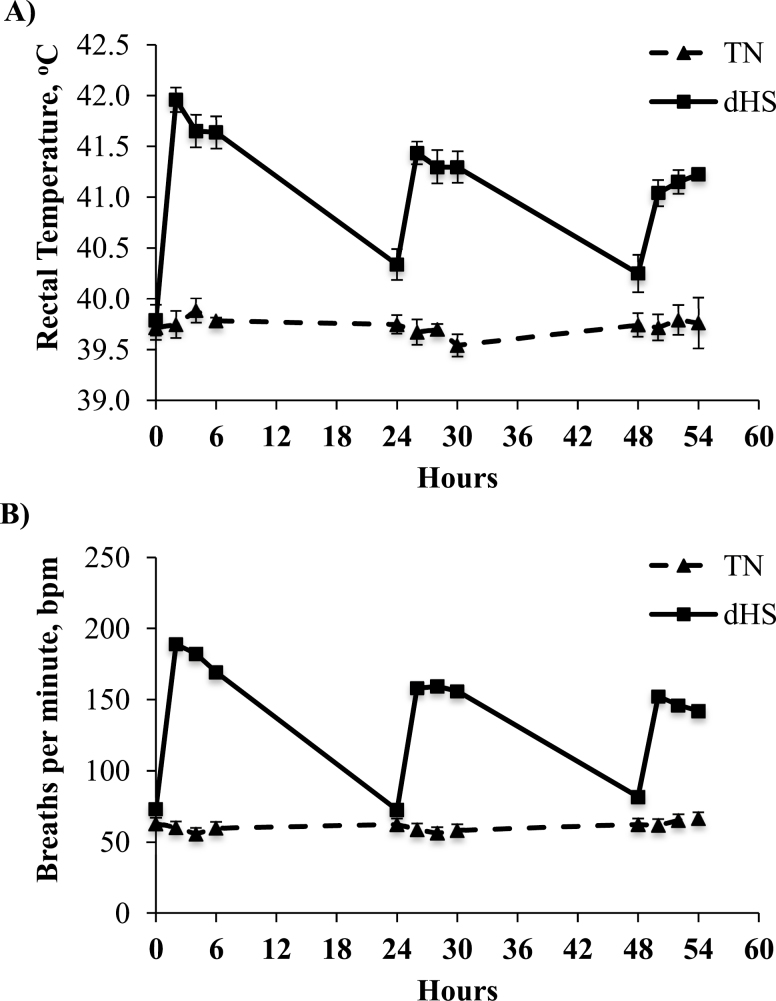
Time course effect of 3 d dHS (6 h at 38°C, 40–60% humidity followed by 18 h at 32°C, 40–60% humidity) or constant TN conditions (28°C; 40–60% humidity) on pig (A) rectal temperature and (B) respiration rates (breaths per min, bpm). *n* = 24/treatment/time point.

No time by treatment interaction was reported for Tr over the 3-d challenge period (*P* = 0.129; Figure 1A). However, there was a significant time effect (*P* < 0.001), and dHS tended to increase Tr compared with TN pigs (41.1 vs. 39.7^o^C, *P* = 0.053). A significant treatment by time interaction and time effect was reported in pig respiration rates (*P* < 0.001; Figure 1B). Furthermore, similar to Tr, respiration rates, overall 3-d respiration rates were increased almost 2.5-fold by dHS compared with TN pigs (60 vs. 140 bpm; *P* < 0.001).

As expected, during the 28-d acclimation period, no differences were reported between treatments in ADG, ADFI, and G:F (Table 2, *P >* 0.10) as both pig groups were reared under identical TN conditions. However, during the 3-d environmental challenge period, dHS pigs had reduced feed intake (30%; *P* < 0.05; Table 2) compared with their TN counterparts. dHS also significantly reduced ADG (85%; *P* < 0.05) and end BWs (7%, *P* = 0.006) compared with TN pigs (Table 2). This translated into a significant reduction (75%, *P* = 0.014) in the 3-d G:F in our dHS-challenged pigs (Table 2).

**Table 2.** Overall growth performance in TN and dHS pigs

Parameter	TN†	dHS†	SEM	*P-value*
Pre-challenge period (0–28 d)				
ADG, kg/d	0.49	0.48	0.017	0.552
ADFI, kg/d	0.65	0.64	0.022	0.838
Gain: Feed	0.76	0.75	0.015	0.393
Environmental challenge (3 d)	TN†	dHS‡	SEM	*P-value*
Start BW, kg	21.9	21.5	0.58	0.423
End BW, kg	23.4	21.8	0.57	0.006
Delta BW, kg	1.47	0.28	0.295	<0.001
ADG, kg/d	0.49	0.07	0.098	<0.001
ADFI, kg/d	0.75	0.54	0.065	0.003
Gain: Feed	0.56	0.14	0.163	0.014

†Constant TN conditions (28°C; 40–60% humidity), *n* = 24 pigs.

‡dHS for 6 h at 38°C (40–60% humidity) and 18 h at 32°C (40–60% humidity), *n* = 24 pigs.

### Ex vivo intestinal integrity and blood endotoxin and LBP level concentrations

**Figure 2.**
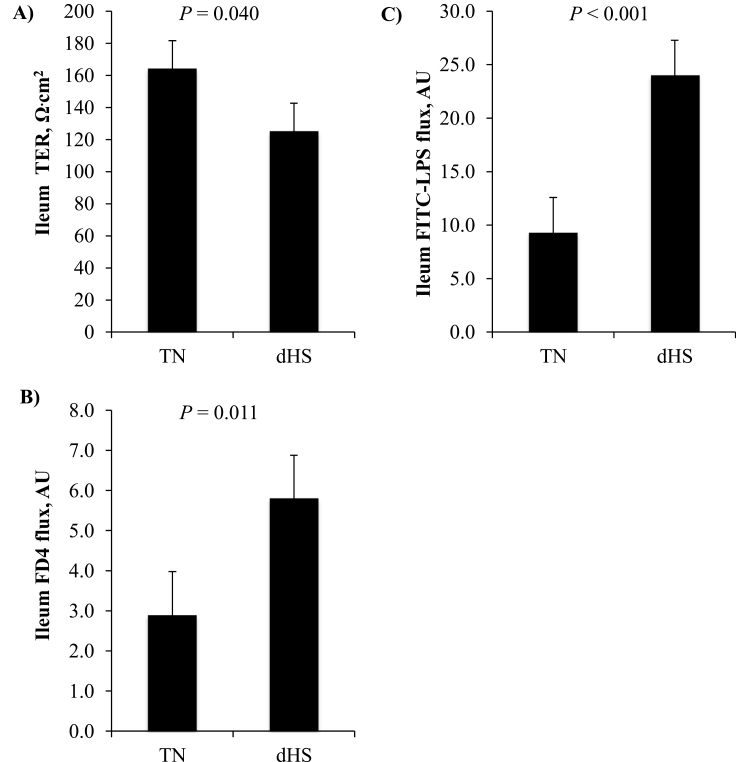
The effect of dHS or TN conditions on ileum integrity. (A) TER, (B) FITC–dextran 4.4 kDa (FD4) flux, and **(C)** FITC–LPS flux. Pigs (*n* = 24) were dHS (6 h at 38°C, 40–60% humidity followed by 18 h at 32°C, 40–60% humidity) for 3 d or reared under constant TN (28°C; 40–60% humidity; *n* = 24 pigs). Ileum segments were assessed for integrity in modified Ussing chambers ex vivo.

Ex vivo assessment of pig ileum integrity via modified Ussing chambers after 3 d of climate challenge showed that TER was significantly reduced (*P* = 0.040) by 25% compared with the TN ileum segments (Figure 2A). Furthermore, macromolecule permeability as assessed by the FD4 flux was significantly increased by 200% compared with the TN ileum tissue (*P* = 0.011; Figure 2B). This decrease in small intestinal integrity also resulted in a 240% increase in the FITC–LPS flux across the ileum segments, measured using the Ussing chambers (*P* < 0.001; Figure 2C).

**Figure 3.**
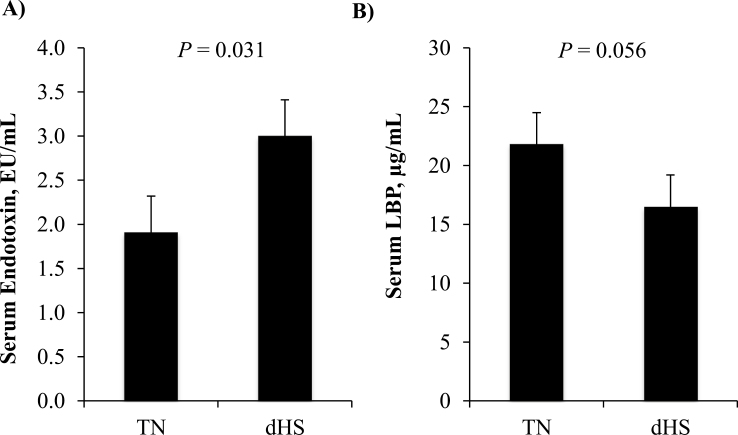
The effect of dHS or TN conditions on circulating serum (A) endotoxin and (B) LPS-binding protein (LBP) concentrations. Blood samples were collected from pigs after they were dHS (6 h at 38°C, 40–60% humidity followed by 18 h at 32°C, 40–60% humidity) for 3 d or reared under constant TN (28°C; 40–60% humidity). *n* = 24 pigs per treatment.

Three days of dHS also increased circulating concentrations of serum endotoxin (Figure 3A). Compared with the control serum, dHS increased serum endotoxin by 150% (*P* = 0.031). Accompanying this increase, there was a tendency (*P* = 0.056) towards a reduction in serum LBP concentrations in dHS pigs (Figure 3B).

### Protein abundance

Protein abundance of HSP 70 and HIF-1α in the ileum and skeletal muscle are shown in Table 3. As expected, Ileum HSP 70 protein abundance was increased (180%, *P* < 0.01) due to dHS environmental treatment compared with control ileums. However, HIF-1α protein abundance in the ileum was not different (*P* = 0.44). In the LD skeletal muscle, dHS did not alter either HSP 70 or HIF-1α protein abundances compared with TN muscle samples (*P* > 0.10).

**Table 3.** Protein abundance of heat shock and hypoxia markers in the ileum and LD muscle of pigs dHS for 3 d.

Parameter	TN^1^	dHS^2^	SEM	*P-value*
Ileum				
HSP70^3^, AU	0.71	1.30	0.126	<0.01
HIF-1α^4^, AU	0.82	0.87	0.154	0.44
*LD*				
HSP70^3^, AU	0.80	0.98	0.161	0.28
HIF-1α^4^, AU	1.07	1.16	0.165	0.45

^1^Constant thermal neutral conditions (TN; 28°C; 40–60% humidity)

^2^Diurnal heat stress (dHS) consisting of 6 h at 38°C (40–60% humidity) and 18 h at 32°C (40–60% humidity)

^3^Heat shock protein 70

^4^Hypoxia inducible factor 1α

n=10 pigs per treatment

### Blood metabolites and inflammatory markers

Blood metabolites and markers of inflammation are reported in Table 4. There was no main effect of treatment on blood glucose concentrations (*P* > 0.05). However, serum NEFA and insulin concentrations were significantly reduced by dHS compared with the TN pigs by 30 and 41%, respectively, *P* < 0.05 (Table 4). There was also a tendency for BUN to be decreased in 3-d dHS pigs compared with the control (14%, *P* ≤ 0.01). sPLA2 was increased due to environmental dHS treatment (*P* < 0.010). Three days of dHS significantly decreased (23%) serum TNF-α concentrations compared with TN pigs. Serum IL-1β was not detectable in these pigs, regardless environmental treatment. Serum haptoglobin concentrations tended to increase due to dHS (138%; *P* = 0.06; Table 4).

**Table 4.** Three day diurnal heats stress effects on pig blood metabolite and inflammation markers

Parameter	TN^1^	dHS^2^	SEM	*P-value*
Glucose, mg/dL	97.2	89.9	9.15	0.479
NEFA^3^, mmol/L	75.6	53.0	6.59	<0.010
Blood urea nitrogen, mg/dL	14.28	12.31	1.076	0.100
Insulin, ng/dL	0.12	0.07	0.022	0.020
sPLA2^4^, µmol/min/ml	11.73	12.11	0.288	<0.010
Tumor necrosis fatcor-α, pg/ml	139	107	14.8	0.040
Interleukin-1β, pg/ml	N.D	N.D	-	-
Haptoglobin, mg/ml	0.47	0.65	0.088	0.060

^1^Constant thermal neutral conditions (TN; 28°C; 40–60% humidity), n=24 pigs.

^2^Diurnal heat stress (dHS) consisting of 6 h at 38°C (40–60% humidity) and 18 h at 32°C (40–60% humidity) n=24 pigs.

^3^Non-esterified fatty acids

^4^Secretory phospholipase A2 activity

N.D. not detected

## DISCUSSION

Due to pigs incapacity to dissipate heat and rapidly adapt to high thermal temperatures, modern, fast-growing, lean domestic pigs can be highly susceptible to heat stress if raised in conditions above its upper critical temperature (Renaudeau et al., 2010). This heat stress results in a reduction in pig performance and, if severe enough, even leads to mortality (Renaudeau et al., 2011). In recent years, many pig studies have utilized a constant elevated temperatures with varying durations to assess how heat stress alters a growing pigs physiology (Pearce et al., 2013a; Boddicker et al., 2014; Sanz Fernandez et al., 2014; Johnson et al., 2015b; Morales et al., 2016a). However, to mimic more closely sub-tropical and temperate region summer heat cycles, some researchers have utilized a more realistic dHS model on growing pigs (Patience et al., 2005; Johnson and Lay, 2017; Kumar et al., 2017; Liu et al., 2017). As such, the current study aimed to characterize the effects of a 3-d dHS model on nursery-grower pig performance, intestinal integrity, and endotoxemia.

In the present study, pigs exposed to 3 d of dHS had an increase in rectal temperature and respiration rates. During the night hours when environmental temperatures were lower, these dHS animals were not able to lower their body temperature to the same level as their TN counterparts. This is in agreements with 2- to 8-d dHS study in pigs that have been reported by Liu et al. (2016, 2017). As expected, both ADG and FI were decreased due to environmental temperature. The magnitude of reduction in feed intake is comparable with those observed in previous experiments by our group with constant heat stress (Pearce et al., 2013c; Pearce et al., 2013d; Sanz Fernandez et al., 2014). This reduction in feed intake is a highly conserved response among species (Collin et al., 2001; Baumgard and Rhoads, 2012).

Liu et al. (2016, 2017) have examined the effects of vitamin and mineral supplementation on heat stress-induced intestinal injury and oxidative stress in pigs. These studies utilized a similar dHS model to describe herein for either 2 or 8 d in duration. Liu et al. (2016) show that dHS induced a significant increase in pig small intestinal permeability (increase FD4 flux and reduced TER values) compared with the thermal neutral pigs. These heat-stressed pigs also had a reduction in performance. This is also in agreement with work that we have previously published in growing pigs reared in continuous high ambient temperatures for 6 h (Pearce et al., 2014) or 1–7 d (Pearce et al., 2013a, 2014, 2015), in which we observed a significant reduction in feed intake, growth, and intestinal integrity.

Growing pigs under heat stress have altered intestinal function and integrity. Reductions in intestinal integrity are in part driven by changes in blood flow due to hyperthermia leading to intestinal hypoxia, barrier dysfunction, and oxidative stress (Hall et al., 2001; Lambert et al., 2002). However, heat stress has been shown to have marginal effects on amino acid digestibility and endogenous loses (Morales et al., 2016a, 2016b), but can improve intestinal epithelial glucose transport (Pearce et al., 2013b). As expected, multiple aspects of ileum intestinal integrity deteriorated due to dHS treatment, including lower TER in the increased FD4 and FITC–LPS flux. Although not assessed in the current study, this could be a result of tight junction protein remodeling or increased villous autolysis (Pearce et al., 2014, 2015).

Although, in the current model, we utilize a less severe heat stress temperature for only 6 h a day, we hypothesized that causing ischemia and reperfusion of blood flow to the intestine each day would potentially be more harmful. In the current experiment, we observed an increase in heat shock proteins in the ileum, but not the muscle, due to dHS. Previously, we have demonstrated increases in heat shock proteins in multiple tissues due to HS, but this is highly time-dependent (Pearce et al., 2013a, 2013b, 2014, 2015). However, HIF-1α, a marker of hypoxia, did not increase due to treatment in the ileum or muscle. Altogether, these data again indicate that the gastrointestinal tract is a major organ affected by heat stress.

We have previously reported an increase in circulating endotoxin accompanying the decrease in intestinal integrity (Pearce et al., 2014; Sanz Fernandez et al., 2014) in pigs under continuous heat stress. In agreement with these studies, compared with TN control pigs, 3 d of dHS increased ex vivo ileum mucosal to serosal FITC–LPS flux by 240% and serum endotoxin concentrations by 150%. Endotoxin or LPS is a potent immune stimulator that induces inflammation (Weber and Kerr, 2008; Mani et al., 2012) and antagonized protein synthesis (Orellana et al., 2002; Kimball et al., 2003; Orellana et al., 2004) and digestibility (Rakhshandeh and de Lange, 2012; Rakhshandeh et al., 2012) in pigs.

Heat stress has also been shown to alter metabolism in some species. In the current study, blood glucose was not altered due to heat stress conditions, and results in glucose concentrations in the blood are highly variable amongst heat stress studies, depending on the model and animal. Animals on a lower plane of nutrition are typically hypoinsulinemic, which is a highly conserved response. Previous heat stress studies in cattle (O’Brien et al., 2010; Wheelock et al., 2010), and pigs (Sanz Fernandez et al., 2015), indicate that heat-stressed animals have higher circulating insulin concentrations and sensitivity compared with pair-fed TN counterparts. In the current study, insulin concentrations were decreased due to dHS compared with TN pigs; however, animals were not on a similar plane of nutrition as indicated by varying ADFI.

Systemic inflammatory responses via the actions of endotoxin and pro-inflammatory cytokines have been proposed and reported as a hallmark of heat-induced tissue injury in humans and rodents (Leon, 2007; Leon and Helwig, 2010). Immunologically, TNF-α, a pro-inflammatory cytokine, was decreased in the blood of the dHS pigs compared with their TN counterparts. This is in agreement with our previous continuous heat stress work in pigs in which we have repeatedly shown TNF-α, IL-8, and IL-1β concentrations and protein expression to be significantly reduced compared with thermal neutral pigs (Pearce et al., 2013b, 2014, 2015). This may be an attempt to by-pass the initial immune response to prioritize the acute phase response. This is aided by the fact that in the current study, dHS pigs tended to have increased circulating concentrations of haptoglobin, an acute phase marker, and this has previously been reported in heat-stressed pigs (Pearce et al., 2013b, 2014, 2015). Intriguingly, secretory phospholipase A2 has been shown to promote inflammation by increasing production of arachidonic acid and other fatty acids. Here we show that dHS causes an increase in circulating sPLA2, but that diet does not affect it. This is somewhat contrary to our cytokine data, but further inflammatory markers need to be tested to determine what is occurring with the immune response. Furthermore, the significant reduction in proinflammatory cytokines, particularly TNFα, could be explained as a tolerance and survival mechanism. Tumor necrosis factor has been shown as a marker of endotoxin tolerance and dramatically reduces following an LPS challenge in tolerized animals (Mathison et al., 1990). This is in contrast to its elevated increase in blood concentrations following first recognition of LPS in challenges pigs (Webel et al., 1997; Gabler et al., 2008).

Undernourished animals mobilize adipose tissue, as a glucose-sparing mechanism to prioritize protein accretion. However, heat-stressed animals do not appear to increase adipose tissue lipolysis (Bobek et al., 1997; Wheelock et al., 2010; Pearce et al., 2013a). In the current study, dHS animals had lower circulating NEFA concentrations, which fit the previously mentioned models. Continuously heat-stressed pigs also have increases in muscle proteolysis (Pearce et al., 2013a; Cruzen et al., 2015a, 2015b); however, under the current conditions, circulating BUN, a marker of nitrogen in the blood, is decreased due to environmental treatment.

Altogether, the data herein demonstrated that dHS over 3 d was sufficient to antagonize pig performance compared with pigs maintained under TN conditions. Furthermore, this model of climate stress reduced small intestinal integrity as demonstrated by decreased ex vivo ileum TER, increased serosal to mucosal FD4 macromolecule flux, and increased serosal to the mucosal flux of FITC–LPS. This reduction is ileum integrity after 3 d of dHS as resulted in dHS pigs having higher circulating concentrations of endotoxin and altered blood metabolite and inflammation profiles. These pre- and post-absorptive effects of dHS may explain why pigs have reduced performance over the summer months.
